# Extra-Peritoneal Suppuration in Perforating Appendicitis

**Published:** 1896-03

**Authors:** J. Lacy Firth

**Affiliations:** House-Surgeon to the Bristol General Hospital


### EXTRA-PERITONEAL SUPPURATION IN PERFORATING APPENDICITIS.

BY

J. Lacy Firth, M.D., M.S. Lond., F.R.C.S. Eng.,

*House-Surgeon to the Bristol General Hospital.*

---

The truth of the statement that the vermiform appendix is a totally intra-peritoneal structure appears to have been satisfactorily demonstrated. There are, however, numerous published statistics which appear to show that a portion of the appendix may be uncovered by peritoneum in a natural state of the parts. Fowler writes : " The completeness with which this [the peritoneum] covers the organ varies greatly. . . . The latter [partially covered] condition was found in more than one-quarter of the observations upon the subject which have been recorded, including those of Maurin, who, in the examination of 112 subjects, found the appendix in the entire number completely surrounded."[2] But other observers have had as uniform results as Maurin, and of the same kind. Kelynack in his monograph, based upon more than 200 examinations, says : " In practically all our cases the appendix was a truly intra-peritoneal structure."[3] Treves is equally definite. Hawkins

[1] *St. Barth. Hosp. Rep.*, 1874, x. 206. [2] *A Treatise on Appendicitis*, 1894, p. 22.
[3] *The Pathology of the Vermiform Appendix*, 1893, p. 41.

speaks of an extra-peritoneal appendix as "an anatomical impossibility";[1] pointing out, however, that the appendix may be closely bound down to the peritoneum lining the iliac fossa.

Ferguson[2] speaks of 77 specially interesting cases out of a total of 200 in which he made *post-mortem* examinations. In these "the appendix was so placed and covered by peritoneum that its perforation would open into the sub-peritoneal tissue, and establish a diffuse form of cellulitis." In all these cases the position was retro-cæcal. But Ferguson does not categorically state that in these 77 cases the appendix was only partially covered by peritoneum, though it is obvious that he regarded the appendices in them as being in specially close relationship with the sub-peritoneal tissue. A retro-cæcal position of a completely invested appendix has been repeatedly observed. Ferguson's statements appear, therefore, lacking in precision in this particular, and they do not seriously oppose the view that the normal anatomical arrangement is that of complete peritoneal investiture.

There have been curious and interesting oscillations of opinion as to the situation of appendicitic abscesses. Some of the earlier writers on the subject, notably Sands, held that they were all primarily extra-peritoneal. Later writers seem to doubt the existence of primary extra-peritoneal abscesses resulting from appendical perforation. Published records show, however, that although peritoneal abscesses are much the more frequent, yet occasionally extra-peritoneal abscesses result without peritonitis. These extra-peritoneal collections of pus are described by Fowler as being bounded posteriorly by the iliac fascia, anteriorly by the transversalis fascia, and above by the peritoneum, which is stripped off the boundaries mentioned and pushed upwards. In a case described in this paper, in which a necropsy was made, the abscess was not bounded behind by the iliac fascia; it was beneath that structure. There appear, therefore, to be three situations in which the abscesses may be produced: (1) in the peritoneum, (2) in the subserous tissue, and (3) in the substance of the ilio-psoas muscle; and an abscess

[1] *On Diseases of the Vermiform Appendix*, 1895, p. 19.
[2] *Am. J. M. Sc.*, 1891, ci. 61.

beginning in any one of these positions may penetrate secondarily into the others.

Talamon quotes statistics which illustrate the relative frequency of the intra- and extra-peritoneal forms of suppuration. He mentions Weir as having in 100 *post-mortem* examinations found 85 cases of peritonitis, 4 of extra-peritoneal suppuration, and 9 of mixed intra- and extra-peritoneal inflammation. Similarly Maurin found in 94 fatal cases 80 of peritonitis, 5 of extra-peritoneal suppuration, and 9 mixed examples. Talamon asks if it is not fair to assume that in some of the cured cases of appendicitis the inflammation has been localised in the cellular tissue? The percentage of cases of extra-peritoneal suppuration in the above lists is 14.9, and of extra-peritoneal suppuration without peritonitis 4.6.

Ferguson asserts that if perforation had occurred in his 77 retro-cæcal appendices, extra-peritoneal suppuration would have resulted. That would have yielded a percentage of 38.5 in 200 cases. The discrepancy in the two computations shows the incorrectness of Ferguson's assumption, and extra-peritoneal suppuration in all probability would not have occurred half as often as he maintains. Many intra-peritoneal abscesses simulating renal and hepatic abscesses have been described and shown to have resulted from perforation of retro-cæcal appendices. The correct explanation of the origin of a primarily extra-peritoneal abscess in appendicitis is no doubt that of Hawkins. He says: "When the appendix is adherent to the iliacus-peritoneum, a perforation of it on the adherent side may lead directly to inoculation of the retro-peritoneal tissue, so that a large abscess may form in the iliac fossa with little or no affection of the peritoneum." [1] The adhesion of the appendix to the iliac peritoneum may be natural or the result of previous pathological processes. The secondarily extra-peritoneal abscesses arise when the peritoneal abscesses cause sloughing and perforation of the parietal peritoneum in contact with them. As Fitz states, within three weeks of this event the iliac muscle may be destroyed and the ilium laid bare. The relative infrequency of occurrence of these extra-peritoneal

[1] *Op. cit.*, p. 92.

abscesses makes the following two cases of special interest. The sequence of events and the pathology of the second case must of course remain doubtful, as the patient recovered; but the case seems to be remarkably similar to the first one, of which the pathology is clearer. The only analogous cases, an account of which I have had an opportunity of seeing, are two mentioned by Kelynack.[1]

CASE 1.—On June 21st, 1895, a labouring man, T. W., aged 72, was admitted to the Bristol General Hospital complaining of a swelling in the right groin. Five weeks before, when returning home from work, he had been seized with pain in the right thigh, and had since then kept his bed. The swelling of which he complained had gradually formed in that time. For a week he had had severe cough. There was no history of rigors, vomiting, or abdominal pain. It was impossible to obtain a more complete account of our patient's illness than the above, he himself being too ill and old to give it, and his wife too intoxicated and indifferent.

The man was in an extremely feeble state on admission. He was sallow, with sunken eyes, and dry, brown, and cracked tongue. Pulse 92, small and weak. Respirations 32. There were large bubbling râles and rhonchi heard all over the chest. There was a huge fluctuating swelling in the upper third of the thigh, the inner border being at the line of the femoral artery. This swelling extended upwards beneath Poupart's ligament into the iliac fossa, and outwards in that region to midway between the crest of the ilium and the last rib. The abdomen being moderately flaccid, the swelling could be further traced upwards near the middle line to the level of an inch above the umbilicus. This uppermost portion of the swelling was deeply situated; but the lower part near Poupart's ligament was superficial, and where superficial in the iliac fossa and in the thigh yielded high-pitched resonance when percussed and audible bubbling when pressed upon.

On the day after admission, Mr. Pickering opened the abscess with two incisions—one near the middle line of the thigh just below Poupart's ligament, the other on the outer border of the thigh. The contents were brownish fæcal-smelling pus and gas. The cavity was felt to extend inwards under the external iliac artery to the brim of the pelvis; upwards its limits could not be reached. It was freely douched with carbolic lotion (1 in 40); and as hemorrhage was free, sponges, thoroughly dusted with iodoform, were packed into the iliac portion. The operation did not improve the patient's condition, and he gradually sank, dying at midnight on the 23rd.

*Necropsy.*—There was no peritonitis present. There was an adhesion of an omental cord to the brim of the pelvis near the symphysis pubis. The cæcum was natural. The vermiform appendix was not obvious at first sight. On careful inspection, and after lifting up the cæcum, it was found passing downwards. It was firmly fixed to the front wall of the abscess. The direction changed to an outward one two inches from the cæcum. This outer portion was half an inch long. On laying open its canal, it was found to end in the abscess cavity. The opening into the abscess was on the adherent posterior surface of the appendix at its termination, was roughly circular, with a diameter of about a

[1] *Op. cit.*, pp. 114–5.

fifth of an inch, and the mucous membrane around the aperture was obviously ulcerated. The abscess had largely destroyed the substance of the iliacus muscle, and a narrow portion extended upwards in the psoas sheath to the level of the hilum of the kidney. The front wall of the abscess in the iliac region was extremely thin, and no differentiation between iliac fascia and peritoneum could be made, these two layers appearing to be intimately blended.

Case 2.—On November 8th, 1894, a signalman, W. M., aged 26, was admitted to the Bristol General Hospital complaining of pain and swelling in the right groin, and with the following history: Ten weeks before admission to the hospital he began to suffer with slight abdominal pain (hypogastric). A day or two later, when bathing at Weymouth, he felt pain in the right iliac region in striking out with the corresponding leg. On the next day the pain was still considerable, and he did not feel as well as usual in himself and his appetite was bad. He returned to work, however, after the few days' holiday he had had, and continued working for two days. On the third day, when at work signalling with his right leg forward, he was again seized with severe pain in the same region (iliac). He walked home, and then had vomiting and diarrhœa. The symptoms persisting at the end of a week, he took to bed and remained there for four weeks. The diarrhœa subsided under treatment in a few days; but his temperature from the time he took to bed for three weeks was constantly between 101° and 103°. The temperature then became normal; and at the end of six weeks he returned to work, having no pain, no abdominal swelling, and no lameness. After two days' work pain returned. It was now in the groin, and led patient to walk with a limp. He again took to bed, and his temperature rose to 102°. The thigh became flexed at the hip, and both the leg and thigh were œdematous, and a swelling gradually formed in the groin. The bowels were regular. There was no abdominal pain, no vomiting, and no shivering. At the end of four weeks he was admitted to the hospital.

He was then pale and sallow in appearance. Temperature 101° in the evening. The right thigh was flexed, forming an angle of 45° with the plane of the bed. Rotation, abduction, and adduction at the hip were well within a limited range; flexion quite free, extension being most of all limited. No pain was elicited when the femur was jarred or moved to a moderate degree. Palpation revealed dense induration extending transversely immediately above Poupart's ligament and outwards from the middle point of that ligament to the middle of the crest of the ilium, past the anterior space, and rising upwards about one and a half inches. This swelling was continuous, with a less marked swelling in the outer part of Scarpa's triangle.

Ten days after admission, palpable gurgling was detected in the swelling, and fluctuation. On the twelfth day Mr. Pickering made two incisions into the swelling, one above and one below Poupart's ligament. Extremely fœtid pus escaped, and a small quantity of gas. On the second day after the operation the temperature dropped to normal, and remained normal until the patient's discharge, when the wounds had healed. When last seen, on July 5th, 1895, the patient was well in all respects, and had been at work for five months.

It will be seen that both the cases were under the care of Mr. C. F. Pickering, and I have to thank him for kindly allowing me to publish them.

